# Pharmacovigilance in oncology

**DOI:** 10.1007/s11096-018-0706-9

**Published:** 2018-08-01

**Authors:** Paolo Baldo, Giulia Fornasier, Laura Ciolfi, Ivana Sartor, Sara Francescon

**Affiliations:** 1 Pharmacy Unit, CRO Aviano IRCCS, National Cancer Institute, Aviano, Italy; 2 Scientific and Patients’ Library, CRO Aviano IRCCS, National Cancer Institute, Aviano, Italy; 3 Scientific Direction, Clinical Trial Office Unit, CRO Aviano IRCCS, National Cancer Institute, Aviano, Italy

**Keywords:** Adverse drug reactions, Cancer, Cytotoxic chemotherapy, Neoplasms, Oncology, Pharmacovigilance, Radiotherapy, Safety, Targeted therapy, Toxicity

## Abstract

*Background* Side effects of cancer therapy are one of the most important issues faced by cancer patients during their illness. Pharmacovigilance, namely the science and activities aimed at monitoring the safety of drugs, is particularly important in oncology, due to the intrinsic biologic toxicity of antineoplastic agents, their narrow therapeutic windows, and the high doses and rigid timing of treatment regimens. *Aim of the review* To identify the main issues in carrying out an effective pharmacovigilance activity in oncology. *Method* We searched PubMed for articles about pharmacovigilance in relation to chemotherapy, radiotherapy and targeted therapy for cancer, using MeSH terms and text words. We also searched Embase, CINAHL, Scopus, Micromedex, the Cochrane Library, two pharmacovigilance databases and the gray literature for articles published in 2012–2018. Overall, 137 articles were considered potentially relevant and were critically appraised independently by two authors, leading to the inclusion of 44 relevant studies, guidelines and reviews. Another 10 important research reports were included in the review. *Results* Eight critical issues of pharmacovigilance in oncology were identified. These issues pertain to: terminology; range of side effects; targeted therapy and immunotherapy; chemoradiotherapy; generic drugs and biosimilars; drug interactions, pharmacogenetics and polypharmacy; special patient categories; and under-reporting of ADRs. *Conclusion* The importance of pharmacovigilance in oncology must be highlighted with every effort, to improve safety and offer cancer patients every possible help to improve their quality of life during such a critical period of their lives.

**Electronic supplementary material:**

The online version of this article (10.1007/s11096-018-0706-9) contains supplementary material, which is available to authorized users.

## Impacts on Practice

- Pharmacovigilance in oncology helps to prevent, detect and manage drug-induced adverse reactions; it also helps to prevent avoidable medical prescription orders.
- Patients should be informed about the toxicity of conventional systemic anticancer drugs and also of innovative targeted therapies.
- Under-reporting of adverse drug reactions in oncology can be addressed through the use of pro-active forms of pharmacovigilance and by multidisciplinary collaborations.

## Introduction

In oncology, clinical research is regularly producing new drugs to use in chemotherapy protocols. The introduction of new drugs that act on specific molecular targets has led to the expectation of low systemic toxicity. Indeed, the safety profiles of new drugs differ from the typical toxicity patterns of conventional chemotherapy, so patients taking these new drugs must be monitored closely to identify new adverse effects.

Starting from the tragedy of thalidomide in the 1960s, the field of pharmacovigilance has developed into an international superstructure that promotes the surveillance of drugs for human use [1]. Pharmacovigilance is today structured in complex communication systems, registries and databases. It is the fundamental approach for the early detection of new signals of risk for patients taking drugs [2]. Pharmacovigilance involves the detection and spontaneous reporting of adverse drug reactions (ADRs) occurring during drug therapy. It can achieve its goal—the safety of drugs—only if its methods are carefully and continuously applied. For this reason, the complete involvement of all health professionals is required, and patient education and involvement are also necessary [3].

### Aim of the review

This literature review aimed to identify the challenges of an effective pharmacovigilance activity in oncology.

## Methods

### Search strategy

We searched for articles about pharmacovigilance in relation to chemotherapy, radiotherapy and targeted therapy in PubMed, using MeSH terms and text words, and Scopus (Table 1). We also searched CINAHL, Embase, Micromedex, the Cochrane Library, two pharmacovigilance databases (EU-ADR and Lareb [4, 5]) and the gray literature for articles published between January 1, 2012 and June 10, 2018. We only considered controlled clinical trials, reviews and guidelines as potentially relevant to this review; inconsistency between objectives and results, undeclared methodology or undocumented differences between the study protocol and methods actually applied were exclusion criteria.

**Table 1:** Search strategies used in this review

Searching PubMed (using the “Advanced Search” interface) **Chemotherapy–targeted therapy–pharmacovigilance** ((((“targeted therapy” OR Molecular Targeted Therapy[MH] OR target*[ti] OR “Antineoplastic Agents/adverse effects”[Mesh] OR “Antineoplastic Agents/toxicity”[Mesh] OR chemotherap*[tiab]) AND (cancer OR tumor OR tumour OR carcinoma OR lymphoma OR sarcoma OR oncology OR ONCOLOG*[TIAB] OR leukemia)) OR NEOPLASMS/DRUG THERAPY[MH]) AND (pharmacovigilance OR Adverse Drug Reaction Reporting Systems[MH] OR pharmacovigilan*[ti] OR Drug-Related Side Effects and Adverse Reactions[MH] OR adverse[ti] OR toxicit*[ti])) AND 2012:2018[dp] **Radiotherapy–pharmacovigilance** (((radiation[ti] OR “Radiotherapy”[Mesh] OR “Radiation, Ionizing/adverse effects”[Mesh] OR radiotherap*[tiab]) AND (cancer OR tumor OR tumour OR carcinoma OR lymphoma OR sarcoma OR oncology OR ONCOLOG*[TIAB])) OR NEOPLASMS/radiotherapy[MH]) AND (pharmacovigilance OR Adverse Drug Reaction Reporting Systems[MH] OR pharmacovigilan*[ti] OR Drug-Related Side Effects and Adverse Reactions[MH] OR adverse[ti] OR toxicit*[ti]) AND 2012:2018[dp] Searching Scopus (using the “Advanced Search” tools) **Chemotherapy–targeted therapy–pharmacovigilance** (TITLE-ABS-KEY (“targeted therapy” OR target* OR antineoplastic OR chemotherap*) AND TITLE-ABS-KEY (cancer OR tumor OR tumour OR carcinoma OR lymphoma OR sarcoma OR oncology OR oncolog* OR leukemia) AND TITLE (pharmacovigilance OR “Adverse Drug Reaction” OR pharmacovigilan* OR “Side Effects” OR adverse OR toxicit*)) AND NOT ((INDEX (medline)) OR TITLE-ABS-KEY (radiotherap* OR radiation)) AND (LIMIT-TO (PUBYEAR, 2018) OR LIMIT-TO (PUBYEAR, 2017) OR LIMIT-TO (PUBYEAR, 2016) OR LIMIT-TO (PUBYEAR, 2015) OR LIMIT-TO (PUBYEAR, 2014) OR LIMIT-TO (PUBYEAR, 2013) OR LIMIT-TO (PUBYEAR, 2012)) **Radiotherapy** (TITLE-ABS-KEY (radiotherap* OR radiation) AND TITLE-ABS-KEY (cancer OR tumor OR tumour OR carcinoma OR lymphoma OR sarcoma OR oncology OR oncolog* OR leukemia) AND TITLE (pharmacovigilance OR “Adverse Drug Reaction” OR pharmacovigilan* OR “Side Effects” OR adverse OR toxicit*)) AND NOT ((INDEX (medline)) OR TITLE-ABS-KEY (“targeted therapy” OR target* OR antineoplastic OR chemotherap*)) AND (LIMIT-TO (PUBYEAR, 2018) OR LIMIT-TO (PUBYEAR, 2017) OR LIMIT-TO (PUBYEAR, 2016) OR LIMIT-TO (PUBYEAR, 2015) OR LIMIT-TO (PUBYEAR, 2014) OR LIMIT-TO (PUBYEAR, 2013) OR LIMIT-TO (PUBYEAR, 2012))	

Articles in English, French, Italian, and German languages identified by the searches were critically appraised by two authors (PB and LC) independently. Ten references published before 2012 were included as additional sources for completeness of the review.

## Results

**Fig. 1:**
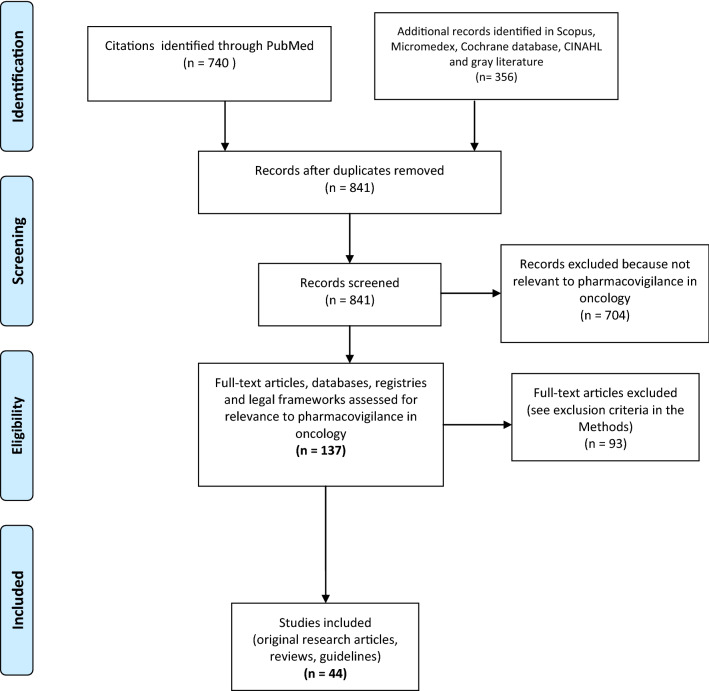
Flow diagram of the selection of studies for this review

Overall, 841 unique records were retrieved by the searches. Records were critically appraised, leading to the selection of 137 potentially relevant articles; finally, 44 relevant studies were included, plus 10 additional references (published before 2012). The PRISMA flow diagram for the search process is presented in Fig. 1. The selected articles revealed eight critical issues relevant to pharmacovigilance in oncology.

### Terminology for classifying adverse events during cancer therapy

Standardized terminology is fundamental for managing the reporting and exchange of data in pharmacovigilance registries. A common lexicon is important to avoid duplicates or the reporting of ADRs that do not correspond to real events. In cancer clinical trials, but also generally in oncology practice, the reporting of adverse events is done according to the Common Terminology Criteria for Adverse Events (CTCAE; version 5, updated November, 2017) [6]. The CTCAE is used by clinicians (and medical records technicians) together with the Medical Dictionary for Regulatory Activities (MedDRA) [7]. The CTCAE system considers the severity of an adverse event, where grade 1 indicates mild toxicity and grade 5 indicates death, while MedDRA focuses on the naming and clinical-pathological classification of the side effects. MedDRA is included in CTCAE and, if applied correctly, enables accurate reporting of ADRs.

To improve the reporting of side effects, the collection of information from the patient’s perspective is useful. For this purpose, the Patient-Reported Outcomes version of the CTCAE (PRO-CTCAE) has been developed [8]. Indeed, there is consensus that the best quality of clinical care and error understanding is achieved by gathering feedback and outcomes from both healthcare professionals and patients themselves [9].

### Side effects of anticancer therapy

A wide range of side effects can be expected from systemic chemotherapy (Table 2) [10–13]. Although most of these ADRs are considered inevitable, decades of clinical expertise in oncology have allowed clinicians to effectively manage them, reducing patient suffering. In this sense, the contribution of pharmacovigilance has been, and continues to be, of paramount importance in recognizing the risks associated with treatments and being able to intervene promptly.

**Table 2:** Adverse effects caused by systemic antineoplastic treatments, and their prevention and management

Adverse effect	Causative agent	Prevention	Management
Cardiovascular toxicity	Anthracyclines, alkylating agents, taxanes, targeted therapies, monoclonal antibodies	Basal assessment of cardiac function (ECHO, LVEF, ECG); evaluation of cardiovascular risk factors and comorbidities	Withdrawal of cardiotoxic therapy; treatment of cardiac dysfunction; ACE inhibitors or beta-blockers should be considered [10]
Cognitive dysfunctionality (chemo-brain, chemo-fog)	Potentially all	Ask patients to report any mental disturbances	No recommendations available
CVC-related complications (infections, thrombosis, extravasation)	All drugs administered in intravenous infusions through CVCs	Monitor patients for CVC-related infections; control regular venous flux and functioning of infusion disposables; train health care personnel	Suspension of intravenous infusions; early surgical procedures to manage extravasation; surgical removal of CVCs; antidotes specific to drugs in extravasation
Dermatological toxicity (skin, hair and nail modifications)	Potentially all systemic cytotoxic treatments (targeted therapies included)	Risk assessment; patient education. Previous treatments can result in cumulative toxicity	Symptomatic treatment based on the grade, type of therapy, and type of cutaneous reactions
Diarrhea or constipation	Antimetabolites, topoisomerase inhibitors, vinca alkaloids, targeted therapies	For diarrhea: fluid intake to prevent dehydration, dietary modifications, nutritional support. For constipation: dietary modifications, fluid intake, physical activity	For diarrhea: antidiarrheal drugs (loperamide), somatostatin analogs if appropriate, probiotics, sulfasalazine. For constipation: laxatives (osmotic or stimulant), opioid antagonists (e.g. methylnaltrexone) for opioid-induced constipation
Fatigue	Microtubule agents	Consider concomitant factors (e.g. pain, anxiety)	Suggest behavioral modifications; provide nutritional, physical and psychological support; consider pharmacological and non-pharmacological approaches
Febrile neutropenia	Taxanes, anthracyclines, antimetabolites, topoisomerase inhibitors, immunomodulatory drugs	Assess the patient’s risk (MASCC score). Use of antibacterial prophylaxis is usually contraindicated. Prophylaxis with G-CSF is recommended if risk is > 20% or if the patient is elderly or has comorbidities	Follow international guidelines (ASCO-ESMO). Patient education and local hospital policies are fundamental
Hormonal impairment, infertility	Cyclophosphamide, taxanes, irinotecan, platinum derivatives	Offer procedures to preserve fertility (e.g. sperm or oocyte banking, shield protection during radiotherapy, ovarian transposition); consider using LH-RH agonists as protection (in women) during chemotherapy	Consider using hormonal replacement therapy and pharmacologic treatment to correct male sexual dysfunction
Infections	Immunomodulatory agents, transplantation	Follow international guidelines (ASCO-ESMO) for prophylaxis (bacterial-viral-fungal); use prophylactic drugs (antibacterials or antivirals) correctly to avoid drug resistance	Accurate diagnosis is essential for choosing a treatment. Anti-infective drugs should be administered to the site of infection (e.g. respiratory tract, head-neck, gastrointestinal, skin, CVC)
Infusion reactions	Potentially all	Risk assessment (e.g. medical history, allergic disorders); premedication with corticosteroids and antihistamines if indicated	Stop or slow the infusion rate, symptomatic treatment
Mucositis	Antimetabolites, methotrexate, cyclophosphamide, platinum derivatives, targeted therapies, taxanes, vinorelbine, 5-fluorouracil	Risk assessment; preventive measures (e.g. oral care, regular dental examinations), nutritional support	Suggest behavioral modifications (avoid alcohol, tobacco, hot foods). Patient education by mean of local hospital guidelines is essential. Use apposite oral solutions to manage symptoms and prevent oral infections [11]
Nausea and vomiting	Anthracyclines and cyclophosphamide in combination, platinum derivatives, azacitidine, bendamustine, ifosfamide, irinotecan, trabectedine	In case of chemotherapy of high emetic risk, give a single dose of 5HT_3_ receptor antagonist, dexamethasone and NK_1_ receptor antagonist before chemotherapy to prevent acute nausea and vomiting	Follow international (ASCO-ESMO-MASCC) and evidence-based guidelines. Antiemetic drugs (corticosteroids, 5-HT_3_ and NK_1_ receptor antagonists, dopamine antagonists, benzodiazepines) must be used in accordance with the emetogenic potential of drugs in the chemotherapy regimen [12]
Neuropathic pain	Microtubule agents (taxanes, vinca alkaloids, eribulin), platinum derivatives	Monitor first infusion, premedicate (corticosteroids or antihistamines), and identify high-risk patients. Previous treatments can lead to cumulative toxicity	Stop infusion of chemotherapy; give nonopioids, at discretion, with or without strong opioids, amitriptyline 25–75 mg/day or gabapentin 300–3600 mg/day [13]
Palmar-plantar erythrodysesthesia (hand-foot skin reaction)	Anthracyclines, antimetabolites, immunomodulatory therapies and targeted therapies	Monitor the patient’s symptoms and behavioral modifications: avoid skin, hand and feet pressure, sun exposure, hot water, friction	Administer oral pyridoxine (up to 150 mg/day); use skin creams (keratolytics or emollients); discontinue or temporarily suspend therapy
Thrombosis	Surgical procedures, non-surgical anticancer treatments	For surgical procedures and implanted accesses, prophylaxis includes low molecular weight heparin, fondaparinux, warfarin	Anticoagulant therapy, low molecular weight heparin, fondaparinux. The use of new anticoagulants in oncology is still under evaluation and is recommended only in select cases

*ASCO*-*ESMO* American Society of Clinical Oncology-European Society for Medical Oncology, *CVC* central venous catheter, *ECHO* echocardiography, *LVEF* left ventricular ejection fraction, *ECG* electrocardiography, *MASCC* Multinational Association of Supportive Care in Cancer, *G*-*CSF* granulocyte-colony stimulating factor, *5*-*HT*_*3*_ serotonin_3_, *NK*_*1*_ neurokinin_1_

### Targeted therapy and immunotherapy

Targeted therapies interfere with specific molecular targets that have a role in tumor growth, progression, and spread. Targeted therapies are usually classified into two main types: monoclonal antibodies (also called immunotherapy, e.g. cetuximab, panitumumab) and small molecule inhibitors (e.g. the tyrosine kinase inhibitors lapatinib and gefitinib). Thanks to their specific mechanisms of action, these drugs have changed cancer treatment [14]. Indeed, these drugs have introduced the concept of tailored cancer treatment by targeting the molecules expressed by cancer. Because of their mechanisms of action, targeted therapies may have less toxicity than conventional chemotherapy [15].

In everyday clinical practice, many side effects can be ascribed to targeted drugs. Although different from the well-known side effects of chemotherapy, these ADRs can severely compromise patients’ quality of life and can even lead patients to discontinue or request a change in therapy. It is important to investigate the toxicity of targeted therapies, because most ADRs included in the Summary of Product Characteristics (SPCs) come from pivotal clinical trials: the list of ADRs identified in these trials is unlikely to be exhaustive, because in clinical trials drugs are being tested under controlled conditions in selected patients [16, 17]. Interestingly, in the FDA Adverse Event Reporting System, monoclonal antibodies are listed among the top ten entries for number of spontaneous ADR reports, with 406,352 records from 2004 to June 5, 2018 (http://open.fda.gov). Compared to conventional systemic chemotherapy, new targeted therapies cause ADRs that are less specific (e.g. gastrointestinal symptoms, altered blood counts, neurologic symptoms, mucositis) but have a more rapid, acute onset because they involve auto-immune reactions and unforeseen inflammatory or hypersensitive responses [18, 19]. Skin toxicity is the most common adverse reaction observed with epidermal growth factor receptor (EGFR) inhibitors and checkpoint inhibitors such as anti-CTLA4 agents (T-lymphocyte-associated antigen 4) and anti-PD-1 agents (programmed cell death protein-1). Immunomediated and acute inflammatory reactions are commonly observed with adoptive cell therapy (CAR-T cell therapy) [20], immunomodulatory therapies [21] and combinations of different agents [22].

### Radiotherapy with chemotherapy

Radiation therapy is a common anticancer treatment. Since radiation treatment is often part of a combined therapeutic strategy (radiochemotherapy), there may be a summation of the incidence of undesirable effects on the patient. Therefore, pharmacovigilance operators require a deep knowledge of the adverse events that can be experienced during radiotherapy, in order to choose the most appropriate management. Adverse events that affect the quality of life of patients include dermatological and oral mucosa toxicity, especially during treatment of head-neck tumors [23], and impairment of fertility or gonadal function [24]. In addition, cardiotoxicity from radiotherapy should be reported, especially in patients with pre-existing risk factors or concomitant cardiotoxic therapy [10, 25]. The diagnosis of a second primary tumor in long-term cancer survivors who had undergone radiotherapy should also be reported [26].

### Generic drugs and biosimilars

The use of generics and biosimilars in oncology is growing exponentially, especially for their lower costs. A generic non-biological drug is defined as a less expensive medicine than the original, off-patent drug, which is equivalent for dose, pharmaceutical form, and route of administration. A biosimilar drug is, instead, a biological medicine with characteristics similar to the original biological drug (the “originator”), although it can have small biochemical differences in the molecular components provided they do not affect the therapeutic activity.

There are important differences in the processes of approval and authorization for use between generics and biosimilars. For generics, the regulation in all developed countries is based on the demonstration of bioequivalence to the branded product (at least 90%) and of similar bioavailability (absorption, distribution). The principles of bioequivalence (for generics) may vary due to the way laboratory tests are conducted, as there is no international consensus or standards proposed by the International Conference on Harmonisation [27]. For biosimilars, each controlling health authority has to ascertain the correspondence of the physicochemical, efficacy and safety characteristics. Biosimilars undergo a longer, more expensive market authorization procedure than generics, even though the biosimilar producer does not have to repeat trial phases 1–4 (which were carried out by the originator) to demonstrate efficacy and safety. (The legislative background is described by Francescon et al. in another article of this *Special Issue.*) Both for generics and biosimilars, the commercial motivations are the reduction of costs and competitive opportunities for a pharmaceutical company after the expiry of patents.

### Drug interactions, pharmacogenetics and polypharmacy

Modern cancer therapy is based on complex treatments involving combinations of chemotherapeutic agents, biologic agents, endocrine agents, growth factors, and targeted therapies. Furthermore, caregivers often add palliative and analgesic therapies, antiemetics and non-pharmaceutical complementary and alternative medicines to help manage ADRs. These complex combinations may increase the number of interactions among drugs or between drugs and other products, including natural ones. These pharmacodynamic and pharmacokinetic interactions can be dangerous to patients and may reduce the benefits of therapy. In addition, since anticancer treatments are increasingly personalized, molecularly targeted therapies (i.e. those that act on specific molecules expressed by particular types of cancer) can have a variety of individual pharmacogenetic responses, which may exacerbate the problem of drug–drug interactions. There may be, for example, genetic variability (polymorphisms) that alters drug transporters or enzymes that metabolize drugs leading to differences in toxicity and efficacy among individuals [28].

The situation of a patient taking multiple medicines concomitantly is called polypharmacy. In general medicine, polypharmacy is defined as a series of medications that have likely been prescribed inappropriately [29]. Hence, in general medicine, polypharmacy is often correctable [30]. In oncology, in contrast, regimens of antitumoral combinations are indicated by guidelines and international consensus and have a strong rationale, which is to attack the tumor with multiple, synergic strategies, while at the same time, to support the body with ancillary therapies. Therefore, in clinical practice, the critical nature of polypharmacy must be referred to the type of setting [31].

### Special patient categories

In oncology, there are several patient groups that require special consideration, even regarding pharmacovigilance. These groups include pregnant women, older patients and children.

#### Pregnant women

It is not rare that cancer presents during pregnancy. Breast cancer is the most frequent concomitant tumor [32], followed by melanoma and hematological malignancies. For these patients, both surgery and systemic chemotherapy are problematic, depending on the stage of gestation and the type of cancer. Anticancer treatment is, however, possible, preferably after week 12. Therefore it is important to motivate pregnant patients. Particular attention is needed to identify individual risk factors and to choose drugs that will have a low impact on the patient’s health and quality of life. Clinicians can rely on the long history of using conventional chemotherapy in these patients, whereas there is less clinical experience using targeted therapies, which present uncertainties about long-term safety for post-partum development and growth. Fortunately, anticancer therapy, when delivered after the first trimester, results in a low percentage (< 5%) of cases of fetal malformations or problems in the developmental age [33]. These data confirm that the international system of pharmacovigilance, starting from its historical origin [34], has produced positive results that now allow us to treat cancer during pregnancy in particularly monitored conditions.

#### Older patients

The prolongation of life expectancy has increased the incidence of cancer in elderly patients over the last few decades. The physical changes that occur with aging (e.g. renal failure, cardiovascular impairment, metabolic problems) often require adjustments in therapeutic regimens and medication dosages. Moreover, these patients have problems with compliance, especially of targeted therapy that is usually taken orally in the home setting.

#### Children

Pediatric oncology is a critical area, for both the impact on patients’ families and the burden of responsibility of caregivers. Clinical–pharmacologic indications in children are different from those in adults, also in cancer care, which often leads to the prescription of drugs in an “off-label” manner. For this reason, pediatric oncologists are not always motivated to report adverse reactions, especially to avoid legal implications or family complaints. Furthermore, many pediatric patients are included in clinical trials, prevalently in non-independent spontaneous research. This situation represents a risk of bias or unbalanced reporting of efficacy of novel or experimental therapies rather than toxicity.

### Under-reporting of ADRs: should we report only severe or new ADRs or all side effects?

Under-reporting of ADRs is common. In oncology, under-reporting is particularly troublesome due to the fact that the underlying clinical conditions of cancer patients can often be confused with ADRs. Furthermore, the toxicity of anticancer drugs is often considered “normal” (or inevitable) and almost all systemic medications have a narrow therapeutic window. Attitudes of health professionals and their level of knowledge about pharmacovigilance are of fundamental importance in establishing the extent of spontaneous reporting [35].

The main causes of under-reporting are common to both general clinical practice and oncology. Documented reasons for under-reporting include a lack of feedback from hospital management [36], fear that reporting ADRs could negatively reflect on one’s competence, and fear of legal controversies or complaints from a patient’s family [37]. Educating health professionals is widely considered a good method to improve reporting, but partnerships with patients’ associations and use of electronic tools (e.g. user-friendly, freely accessible web platforms for reporting ADRs instead of paper forms such as the yellow cards) can also help [38, 39].

The early detection of safety signals is also important, considering the expanded use of accelerated approval licensing paradigms, which are common in oncology and hematology. There has been little consensus among health professionals on whether only new, previously unreported or severe ADRs should be reported or if all observed side effects (which, in oncology, are many) must be reported. Recent European legislation on pharmacovigilance, in effect since 2012 and updated in 2017 (Directive 2010/84/EU; Regulation (EU) no. 1235/2010), has radically changed the definition of “adverse reaction”: now, medical professionals are de facto obliged to report all observations of undesirable events correlated, with high probability, with the use of a drug. In the new EU Directive, even lack of efficacy is considered an ADR, because it could derive, for example—in the most trivial cases—from defective batches or errors in drug administration [40].

## Discussion

Modern pharmacovigilance is the observational strategy through which today we can hope to avoid epidemiological tragedies such as that of thalidomide in the 1960s. In clinical practice, international pharmacovigilance systems structure the spontaneous reporting of the adverse events, which is the only way to generate new risk signals related to the use of drugs. Thanks to the FDA’s Risk Evaluation and Mitigation Strategies program [41], one of the procedures introduced by pharmacovigilance regulations, it has been possible to readapt thalidomide and similar medicines (e.g. lenalidomide, pomalidomide), on the basis of their highlighted therapeutic efficacy, for use in other diseases (e.g. multiple myeloma).

While the toxicity profiles of conventional chemotherapy are well known, and essentially concern the manifestation of defence systems and overall weakening of the body, targeted and immunomodulatory therapies still present unknown aspects and are more based on personalization; consequently they are more likely to cause immune or autoimmune reactions. This review has highlighted eight critical issues, both negative and positive, for the pharmacovigilance of anticancer treatments.

First, while the reasons for under-reporting are well known, lack of knowledge can no longer be used as an excuse to avoid ADR reporting, even in oncology. Additionally, the accelerated or conditional approval of new drugs (by the FDA or EMA) allows the clinical use of anticancer drugs without a clearly defined risk–benefit ratio, often with minimum benefit (e.g. an increase in overall survival of 3 months on average), with an unknown risk of adverse effects in the general population [42–45]. Moreover, while the advent of digital social media favors information sharing, it also increases the possibility that inaccurate or biased information confound the collection of drug safety data from patients [46].

On the brighter side, there is evidence of progress in the correct reporting of ADRs in oncology. For example, the Weber effect (the theory that postulates a peak of reports of ADRs in the second year after marker authorization, followed by a decline) is not significantly present for anticancer drugs [47]. Moreover, the implementation of digital pharmacovigilance systems can improve the quality of life of cancer patients through the prompt reporting of adverse reactions [48, 49]. Scientific societies are showing great productivity in the establishment of guidelines, tools and platforms for the reporting of ADRs in clinical trials and in oncology research [50, 51]. Sponsored clinical trials in oncology do not seem to emphasize the positive effects over the toxicity of anticancer drugs, compared to non-sponsored studies [52].

It is essential to maintain a high level of attention, because many of the side effects caused by new drugs have a rapid, unpredictable onset (as in the case of cytokine release syndrome). If not promptly identified, these ADRs can be potentially life-threatening conditions. Aging and co-morbidities increase the complexity of the problem, making interactions among drugs more likely to compromise the efficacy of, or reduce compliance, to therapy, especially in elderly and pediatric patients. Moreover, the assignment of causality of an adverse effect to a particular drug or pre-existing risk factor is difficult [53]. For this reason, complete information about a patient’s predisposing risk factors is important in avoiding suffering and improving quality of life.

## Conclusion

The spontaneous reporting of ADRs is an important task for clinical pharmacists and other health professionals. This task is most efficient when standardized terminology is used (e.g. CTCAE and MedDRA) and when the data are correctly deposited in international pharmacovigilance registers.

Finally, the phenomenon of under-reporting requires great attention in oncology, especially because oncologists often consider adverse reactions caused by cytotoxic drugs as a “normal” phenomenon, or may delegate the reporting to nononcologists or nurses [54]. Moreover, the underlying clinical conditions of cancer patients often make it difficult to distinguish ADRs from symptoms of cancer.

Efficient management of spontaneous ADR reports is essential to monitor drug safety in oncology, where pharmacotherapy is de facto affected by a high prevalence of drug-related complications and a narrow therapeutic window. Clinical pharmacists qualified in pharmacovigilance have great responsibilities to promote safety, carefully follow cancer patients in treatment, and support educational initiatives. The importance of pharmacovigilance in oncology must be highlighted with every effort, to improve safety and offer cancer patients every possible help to improve their quality of life during such a critical period of their lives.

## Electronic supplementary material

Below is the link to the electronic supplementary material.

Supplementary material 1 (DOCX 14 kb)

Supplementary material 2 (DOCX 31 kb)
