# Supplementary material for: Pharmacovigilance in oncology
Source: Int J Clin Pharm. 2018 Aug 1;40(4):832–41. doi: 10.1007/s11096-018-0706-9 (PMC6132974; doi:10.1007/s11096-018-0706-9)
Supplement: Supplementary file 1 — Supplementary material 1 (DOCX 14 kb) [file 11096_2018_706_MOESM1_ESM.docx]

| **Under-reporting of adverse drug reactions in pharmacovigilance (results reported in Table 3)**  ***a.*** *Search in PubMed*  (((("adverse drug effects" OR "adverse drug event" OR "adverse drug events" OR "adverse drug reaction" OR "adverse drug reactions" OR "adverse drug toxicity" OR Drug-Related Side Effects and Adverse Reactions[MH] OR pharmacovigilan* [ti] OR "Pharmaceutical Preparations/adverse effects"[Mesh] OR ("Chemicals and Drugs Category"[Majr] AND (side effect*[tiab] OR ADVERSE[TI] OR "ADVERSE EVENT"[TIAB] OR "ADVERSE EVENTS"[TIAB] OR safe*[ti] OR adverse reaction*[tiab])) ) AND ("underreport"[tiab] OR "under report"[tiab] OR underreport* [tiab] OR report*[ti] OR Self Report[MH] OR "Adverse Drug Reaction Reporting Systems"[Mesh] OR "adverse drug reaction reporting" OR "adverse drug reaction reporting systems"))) AND (((review[pt] AND "systematic REVIEW") OR "SYSTEMATIC REVIEW"[TI]) OR Cochrane Database Syst Rev[TA]) ) NOT (animals OR pets[mh])  ***b.***  *Search in Embase*  (chemicals and drugs/exp/adverse drug reaction,drug toxicity, drug interaction OR chemicals and drugs-induced :de,ab,ti OR (( chemicals and drugs :de OR chemicals and drugs:tn,ti,ab OR drug:tn,ti,ab OR drugs:tn,ti,ab) AND (adverse drug reaction /exp OR adverse drug reaction :lnk OR adverse:de,ab,ti OR (((side OR undesirable OR unwanted) NEXT/2 (effect* OR reaction* OR event* OR outcome*)):de,ab,ti) OR side effect /lnk OR side effect /exp OR complication /lnk OR complication /exp OR complication*:de,ab,ti OR worsening :de,ab,ti OR case report* :de,ab,ti OR pharmacovigilance :de,ab,ti OR postmarketing surveillance /exp OR drug interaction :lnk OR drug interaction /exp OR toxicity /exp OR drug toxicity :lnk OR toxic*:de,ab,ti OR intox*:de,ab,ti OR safety:de,ab,ti OR poison*:de,ab,ti OR pharmacotox*:de,ab,ti OR neurotox*:de,ab,ti OR cardiotox*:de,ab,ti OR nephrotox*:de,ab,ti OR hepatotox*:de,ab,ti OR immunotox*:de,ab,ti OR immunocytotox*:de,ab,ti OR cytotox*:de,ab,ti OR carcinogen*:de,ab,ti OR cancerogen*:de,ab,ti OR mutagen*:de,ab,ti OR terato*:de,ab,ti OR fatal outcome /exp OR death /exp OR death*:de,ab,ti OR suicide /exp OR suicid*:de,ab,ti OR mortal*:de,ab,ti OR fatal*:de,ab,ti OR risk /exp OR nocebo:de,ab,ti OR lethal concentration /exp OR iatrogenic disease /exp OR fertility /exp OR substance-related disorders /exp OR chemically induced:de,ab,ti OR morbidity :de,ab,ti OR congenital disorder :de,ab,ti OR infertility /exp OR injury /exp))) AND ( human /exp OR human OR m?n OR wom?n OR child OR boy OR girl OR patient*) AND ( drug surveillance program /exp/mj OR report.ti OR underreport*.ti OR under report*.ti ) AND ([cochrane review]/lim OR [systematic review]/lim) NOT [medline]/lim |
| --- |

***Additional Table 1.*** *Search strategy for “Under-reporting and Pharmacovigilance”*
