# Supplementary material for: Pharmacovigilance in oncology
Source: Int J Clin Pharm. 2018 Aug 1;40(4):832–41. doi: 10.1007/s11096-018-0706-9 (PMC6132974; doi:10.1007/s11096-018-0706-9)
Supplement: Supplementary file 2 — Supplementary material 2 (DOCX 31 kb) [file 11096_2018_706_MOESM2_ESM.docx]

| Title of the Study | | Journal/Pub. Year/doi or PUI/e-link | Specific outcomes / Results /Reasons for under-reporting (UR) | Recommendations |
| --- | --- | --- | --- | --- |
| *Goedecke et al*. Measuring the impact of medicines regulatory interventions – Systematic review and methodological considerations | British Journal of Clinical Pharmacology / 2018 /  <http://dx.doi.org/10.1111/bcp.13469> | | 153 studies included in descriptive analysis. No specific outcomes have been described. / Heterogeneity of methods and conduct across the selected studies. | Need of scientific guidance and systematic education and dissemination of results. |
| *Chopra et al*. Data management in pharmacovigilance: Challenges and complexities | Drug Safety / 2017 / <http://dx.doi.org/10.1007/s40264-017-0580-8> | | Not described. | Need of good and transparent quality data documentation and management. |
| *Al Dweik R et al.* Factors affecting patient reporting of adverse drug reactions: a systematic review. | Br J Clin Pharmacol /2017 / doi: 10.1111/bcp.13159 | | 21 studies included. Main cases for UR: unawareness, lack of feedbacks, difficulty in using reporting systems. | Need for clear reporting processes and systems to facilitate patients’ reporting. |
| *Inacio P et al.* The value of patient reporting to the pharmacovigilance system: a systematic review. | Br J Clin Pharmacol / 2017 / doi: 10.1111/bcp.13098 | | 34 studies included. Patient’s reporting brings detailed description of ADRs, making itself complementary or integrative for the reporting made by health professionals. | Need to overcome the gaps that need to overcome the gaps that prevent the full participation of patients in the voluntary reporting of ADRs. |
| *Reumerman M et al*. Is there a need for pharmacovigilance education in our future healthcare professionals? a review | Clinical Therapeutics / 2017/  PUI: L618193026 | | 34 studies included. Ignorance and unawareness about PV reporting systems among future Healthcare professionals | Need to improve educational programs for PV. |
| *Golder S et al.* Reporting of Adverse Events in Published and Unpublished Studies of Health Care Interventions: A Systematic Review. | PLoS Med / 2016 / doi: 10.1371/journal.pmed.1002127 | | 28 studies included. Strong evidence that much of the information on ADRs remains unpublished and that the number and range of adverse events is higher in unpublished than in published versions of the same study. | The inclusion of unpublished data in meta-analyses can reduce the imprecision of estimates of adverse events, and can reduce biases in interpretation and generation of signals for PV. |
| *Bailey C et al.* Adverse drug event reporting systems: A systematic review | British Journal of Clinical Pharmacology / 2016 / <http://dx.doi.org/10.1111/bcp.12944> | | 33 mapped reporting concepts; variability in the data fields used to report ADEs, limiting the comparability of ADE data collected | Need for development of common standardized data set; need to share common concepts and use homogeneous lexicon. |
| *Lee JY et al*. Systematic Review of Adverse Effects from Herbal Drugs Reported in Randomized Controlled Trials*.* | Phytother Res. / 2016 / doi: 10.1002/ptr.5647 | | 244 studies included (111 of a single herb and 133 of multiple herbs) for a total of 15 441 participants. The review describes the most frequently reported adverse events following use of herbs for therapeutic reasons. | Not described. |
| *Ribeiro-Vaz I et al.* How to promote adverse drug reaction reports using information systems - a systematic review and meta-analysis. | BMC Med Inform Decis Mak / 2016 / doi: 10.1186/s12911-016-0265-8 | | 29 projects described in the review. 7/29 were included in a meta-analysis. Interventions resulted in doubling the number of spontaneous reports of individual cases of ADRs. | Need to develop electronic systems which help healthcare professionals in ADR reporting¸ Alternatively, a tool should be proposed to facilitate reporting ADRs. This tool can be promoted by sending emails or through the inclusion of direct hyperlinks on healthcare professionals' desktops. |
| *Atkinson et al.* The association between clinician-based common terminology criteria for adverse events (CTCAE) and patient-reported outcomes (PRO): a systematic review. | Supportive Care in Cancer / 2016 / <https://doi.org/10.1007/s00520-016-3297-9> | | 28 studies included. Great variability of association between CTAE-based reports and PRO-based reports. | Need to develop patient-targeted language and tools to facilitate access to spontaneous reporting of ADRs. The development of PRO-CTACAE tool can be a good example for such tools. |
| *Varallo et al.* Causes for the underreporting of adverse drug events by health professionals: a systematic review. | Revista da Escola de Enfermagem da USP /2014 / <http://dx.doi.org/10.1590/S0080-623420140000400023> | | 29 studies included. The main causes related to underreporting were ignorance (24/29), insecurity (24/29) and indifference (23/29). | Need for continuing education aimed to increase adherence of professionals to PV activities and improve awareness and communication of drug-related risks. |
| *Tarapués M et al.* The webreporting format to report adverse drug reactions in latin-american countries - An affordable way to overcome the underreporting. | Drug Safety /2013 <http://dx.doi.org/10.1007/s40264-013-0087-x> | | Web-reporting observed only in 4 /11 Latin-American countries. | Need to encourage web-reporting and to improve information about PV in websites. |
| *Gonzalez-Gonzalez C et al.* Strategies to improve adverse drug reaction reporting: a critical and systematic review. | Drug Safety / 2013 /doi: 10.1007/s40264-013-0058-2 | | 43 studies included. Great heterogeneity and formal limitations in data collection across studies; low grade of evidence. Multiple interventions seem to have had more impact than did single interventions. | Need for studies with better methodological design, aimed to improve ADRs reporting by health professionals. Multiple, synergic interventions seem to generate better improvements in the ADR reporting rates than a single isolated intervention. |
| *Péron J et al.* Adherence to CONSORT adverse event reporting guidelines in randomized clinical trials evaluating systemic cancer therapy: a systematic review. | J Clin Oncol. / 2013 / doi: 10.1200/JCO.2013.49.3981 | | 325 RCTs reviewed. The methods of AE collection and analysis were highly heterogeneous across studies; the methods of AE collection were reported in only 10% of included studies. | Recommendation to clinicians and trials investigators to adhere to the CONSORT guidelines regarding AE reporting. |
| *Inch J et al.* Patient versus Healthcare Professional Spontaneous Adverse Drug Reaction Reporting | Drug Safety / 2012 / <https://doi.org/10.1007/BF03261977> | | 3 studies included; results showed similarity of serious ADR reporting patterns between patients and HCP; the absolute number of reported cases varied across the studies. | Need for further systematic investigations and comparisons, because the real value of patient ADR reporting to the process of signal generation in PV remains still unclear. |
| *Molokhia M.* et al. Improving reporting of adverse drug reactions: Systematic review. | Clinical Epidemiology / 2009/ PMID=20865089 | | 24 studies included. The use of computerized  interventions combined with reminders and/or prescription card reports seems to improve, in a short to medium term. hospital-based ADR reporting, | Need to combine electronic health data combined with other methods of communication for ADR reporting. |
| *Lopez-Gonzalez E et al.* Determinants of under-reporting of adverse drug reactions: a systematic review*.* | Drug Safety / 2009 / doi: 10.2165/00002018-200932010-00002 | | 45 studies included. Under-reporting resulted associated with medical specialty (76% of studies), ignorance (only severe ADRs need to be reported) diffidence, lethargy, procrastination, lack of interest and personal attitudes of health professionals. | Need to understand if interventions that modify knowledge and awareness are potentially capable to improve attitudes toward reporting ADRs by HCPs. |
| *Phansalkar S et al*. Pharmacists versus non pharmacists in adverse drug event detection: a meta-analysis and systematic review. | American Journal of Health-System Pharmacy / 2007 / doi: 10.2146/ajhp060335 | | 13 studies included. inclusion criteria. The mean of the weighted incidence rate detected by pharmacists was 0.33 ADEs per admission, versus 0.16 ADEs detected by non pharmacists; and a significative difference in ADEs reporting rate (0.23 versus 0.12, respectively). | The meta-analysis highlighted evidence that pharmacists make a salient contribution as reviewers in inpatient ADE interventions. This can improve prevention and management of ADRs. |
| *Hazell L et al*. Under-reporting of adverse drug reactions: a systematic review. | Drug Safety / 2006 / <http://dx.doi.org/10.2165/00042310-200723030-00007> | | 37 studies included from 12 countries. These generated 43 numerical estimates of under-reporting. The review provides evidence of significant and widespread under-reporting of ADRs to spontaneous reporting systems including serious or severe ADRs. | Need to improve reporting such as internet reporting, pharmacist/nurse reporting and direct patient reporting as well as improved education and training of healthcare professionals. |

**Additional Table 2.** Systematic reviews investigating the phenomenon of under-reporting in Pharmacovigilance.

*For the optimized search strategy, see additional Table 1, under-reporting in PV, point* ***a.*** *(Pubmed) and point* ***b.*** *(Embase)*. PUI=Publisher Item Identifier (Embase); DOI=digital object identifier (PubMed); PMID=PubMed identifier (reference number); HCPs=Healthcare professionals; PV=Pharmacovigilance; ADRs=Adverse drug reactions; CONSORT=Consolidated Standards of Reporting Trials; RCTs=Randomized clinical trials
